# Entrustable Professional Activities (EPA) im Modellstudiengang Zahnmedizin „iMED DENT“ am Universitätsklinikum Hamburg-Eppendorf

**DOI:** 10.1007/s00103-023-03803-3

**Published:** 2023-11-14

**Authors:** Guido Heydecke, Christine Mirzakhanian

**Affiliations:** Poliklinik für Zahnärztliche Prothetik, Zentrum für Zahn‑, Mund- und Kieferheilkunde, Universitätsklinikum Hamburg-Eppendorf, Martinistr. 52, 20246 Hamburg, Deutschland

**Keywords:** EPA, Bewertung klinischer Tätigkeiten, Bewertungsformate, Zahnmedizinische Lehre, Kompetenzbasiertes Lernen, EPA, Evaluation of clinical activities, Valuation type, Dental education, Competency-based learning

## Abstract

Im Studium der Zahnmedizin nimmt die Ausbildung klinischer Tätigkeiten einen besonderen Stellenwert ein, da ab dem 7. Fachsemester Behandlungen an Patient:innen durchgeführt werden. Die Kompetenzen eines Zahnarztes müssen zum Antritt des Staatsexamens für die sich daran anschließende Berufsausübung vorliegen. Die zu erlernenden klinischen Tätigkeiten erfordern eine Vorbereitung durch präklinische Übungen und frühen Patientenkontakt schon vor dem 7. Fachsemester sowie den Ausbau der Kompetenzen in einem Schritt-für-Schritt-Vorgehen. Bei der Entwicklung des Modellstudiengangs iMED DENT am Universitätsklinikum Hamburg-Eppendorf wurde hierfür ein Katalog an „anvertraubaren professionellen Tätigkeiten“ (EPA) erstellt. Eine EPA ist definiert als eine anvertraubare klinische Tätigkeit (z. B. Herstellung einer Einzelkrone als festsitzender Zahnersatz), die zum Zeitpunkt der Durchführung ein definiertes Kompetenzniveau erreicht. Innerhalb einer EPA werden die Kompetenzen Theorie, praktisches Geschick und Umgang mit Patient:innen ausgebildet. Das verwendete Bewertungsformat muss sicherstellen, dass die darüber geleisteten Rückmeldungen zur Qualität der durchgeführten klinischen Behandlungsschritte die jeweils relevanten Kompetenzen erfassen. Die Bewertung der Kompetenzen innerhalb der EPA wird mit einem abgestuften Bewertungsschema vorgenommen (A = sehr gut, B = gut, C = schlecht, D = Leistung nicht/unvollständig erbracht). Das Bewertungsschema dient als Feedback-Werkzeug zur Verbesserung des Lernerfolgs und ist gleichzeitig Grundlage für die Bewertung der hochschulinternen Prüfungen.

## Abstract (en)

In dental education, the assessment of clinical activities is especially important, as students start to perform treatments on real patients beginning in the seventh semester. When entering the final exam, students must be capable of carrying out all treatments of a dental professional, as they will exercise their profession as dentists directly after the exam. During dental training, preparation for the performance of dental activities in a clinical context is achieved through preclinical coursework and early patient contact before the seventh semester to enhance competencies. In the course of developing the iMED DENT curriculum of undergraduate dental training at the University Medical Center Hamburg-Eppendorf (UKE), a catalogue of entrustable professional activities (EPAs) has been defined. An EPA comprises a clinical procedure (e.g., fitting of a crown) corresponding to the students’ level of competency at the time of performance. Each EPA includes teaching within the three core competencies: “theoretical knowledge,” “clinical skills,” and “patient communication.” The applied assessment formats have to ensure that the feedback given on clinical performance contains all relevant competencies. The EPAs are evaluated using a graded “ABCD assessment” scheme (A = very good, B = good, C = bad, D = activity was not completed/incomplete). The assessment scheme serves as a tool for feedback to improve learning success and at the same time for evaluation of examinations during the course of studies.

## Einleitung

Der Modellstudiengang iMED DENT wurde mit dem Ziel entwickelt, ein symptomorientiertes Curriculum zu schaffen, das durch die durchgehende Integration klinischer und theoretischer Anteile geprägt wird. Entsprechend ist das zentrale Leitbild des Studiums die themen- und symptombezogene Vernetzung der zahnmedizinischen Disziplinen untereinander ebenso wie die themen- und symptombezogene Vernetzung der zahnmedizinischen Disziplinen mit Grundlagenfächern und klinischen Fächern der Medizin. Hierbei spielen frühe klinische Bezüge durch die anwendungsnahe Präsentation von Grundlageninhalten eine große Rolle [1].

Neben den theoretischen fachlichen Grundlagen sollen ab Studienbeginn manuelle Feinfertigkeiten entwickelt und bis zum 6. Fachsemester in praktischen zahnmedizinischen Kursen am Phantommodell trainiert werden. Durch die Integration „interstudentischer“ klinischer Übungen bereits ab dem 2. Fachsemester wird ein klinischer Patientenkontakt durch nichtinvasive Behandlungsübungen simuliert und somit frühe klinische Erfahrungen ermöglicht, was ein wichtiges Reformziel des Modellstudiengangs iMED DENT darstellt. Dieser „frühe Patientenkontakt“ ist eingebettet in die Kurse am Phantommodell des 2., 3., 5. und 6. Fachsemesters. Im 5. und 6. Fachsemester werden die vorher durchgeführten interstudentischen Übungen durch zahnmedizinische Assistenztätigkeit und Befundaufnahmen bei Patient:innen erweitert und die Studierenden so an die Behandlung ab dem 7. Fachsemester herangeführt.

Zur Vorbereitung auf den Kontakt zu „echten“ Patient:innen im klinischen Studienabschnitt ab dem 7. Fachsemester müssen gemäß den Zielen des integrierten Modellstudiengangs die interpersonellen Fähigkeiten der Studierenden im Hinblick auf ein angemessenes ärztliches Verhalten entwickelt werden. Dies erfolgt longitudinal über das gesamte Curriculum in Modulen, in denen die Kommunikation und ärztliche Gesprächsführung gelehrt werden.

Ein zentraler Bestandteil im Regelcurriculum nach der gültigen Approbationsordnung für Zahnärzte und Zahnärztinnen (ZApprO, ausgefertigt 2019; [2]) wie im Curriculum des hier thematisierten Modellstudiengangs ist die klinische Ausbildung mit Patientenbeteiligung in den integrierten Behandlungskursen. In diesen klinischen Behandlungskursen im 7.–10. Semester werden die für die spätere Berufsausübung notwendigen klinischen Kompetenzen ausgebildet.

In den bisherigen Lehr- und Prüfungsformen wurden viele Prüfungsschritte mit Testaten abgeschlossen. Diese waren mitunter eine reine Bestätigung dafür, dass ein Arbeitsschritt abgeschlossen wurde. Ein intensives inhaltliches Feedback war damit oft nicht verbunden. Insbesondere die Aspekte interpersonellen Verhaltens wie im Beispiel Patientenkommunikation lassen sich in einem solchen Schema nicht gut abbilden. Aus lernpsychologischen Gründen mit dem Ziel einer verbesserten Selbstregulation ist es erforderlich, ein Leistungsevaluations- und Prüfungssystem zu entwickeln, das regelmäßige Rückmeldungen in allen relevanten Bereichen, Theorie, praktische Durchführung und ärztliches Verhalten, ermöglicht und dadurch die Selbsteinschätzung der Studierenden bezüglich ihrer eigenen Leistungen verbessert. Daher wurde im zahnmedizinischen Studiengang iMED DENT ein Modell zur kompetenzbasierten Ausbildung für die klinischen Tätigkeiten der Patientenbehandlungen implementiert. Darin sind anvertraubare professionelle Tätigkeiten (Entrustable Professional Activities), kurz EPA, definiert. Eine EPA entspricht einer zahnärztlichen Handlungseinheit, die zum Zeitpunkt der Durchführung auf dem vorliegenden Fertigkeitsniveau bewertet wird. Eine EPA wird im Hinblick auf die 3 Kategorien *Theorie, praktische Durchführung und ärztliches Verhalten* jeweils mittels „ABCD-Bewertung“ bewertet (A = sehr gut, B = gut, C = schlecht, D = Leistung nicht/unvollständig erbracht) und die 3 Teilnoten nach Abschluss zur Gesamtbewertung zusammengeführt.

In diesem Bericht werden die Ausbildung klinischer Tätigkeiten innerhalb eines EPA-basierten Konzeptes und deren Bewertung mittels ABCD-Bewertungsschemas im Modellstudiengang iMED DENT am Universitätsklinikum Hamburg-Eppendorf (UKE) vorgestellt und ihre Anwendung beschrieben.

## Bewertungen klinischer Tätigkeiten mithilfe von EPA in der Medizin

Bereits 2007 beschrieb ten Cate für den medizinischen Kontext die Lücke zwischen theoretischem Wissen und klinischer Praxis bei Studierenden. Die Überwindung der Grenze von Theorie zu Praxis findet laut ten Cate oft erst nach der ärztlichen Zulassung statt. Er empfiehlt EPA als kompetenzbasiertes Grundgerüst in der postgradualen Ausbildung und in Famulaturen. Hier sollte dem Nachwuchs Verantwortung für eingegrenzte vordefinierte professionelle Tätigkeiten übertragen werden. Die Einhaltung einer korrekten Terminologie für die Anwendung dieses Lehrmodells ist laut ten Cate essenziell. Nach ten Cate beschreibt der Begriff Kompetenz „die Fähigkeit, etwas erfolgreich durchzuführen“. Studierenden sollten diese Tätigkeiten erst dann anvertraut werden, nachdem sie alle aufbauenden, hierfür erforderlichen Kompetenzen für eine erfolgreiche Durchführung der Tätigkeit erlangt haben [3].

Die „Association for American Medical Colleges“ (AAMC) hat aufgrund in der Literatur beschriebener auftretender Leistungsdefizite von Absolvent:innen zu Beginn der postgradualen Assistenzzeit („residency“) im Jahr 2014 verschiedene EPA definiert, die die Fähigkeiten beschreiben, die nach dem Abschluss des Grundstudiums vorliegen müssen. EPA wurden hierfür ausgewählt, da sie eine praktische Herangehensweise an die Bewertung von Kompetenzen in realen Situationen bieten [4].

Die Verwendung von EPA für die postgraduale Ausbildung und Famulaturen in der Medizin ist übertragbar auf die zahnmedizinische Ausbildung. Bereits während des Studiums werden klinische Tätigkeiten durchgeführt und ab dem Staatsexamen müssen die anvertraubaren professionellen Tätigkeiten für die Berufsausübung entwickelt sein, wohingegen im medizinischen Bereich nach der Approbation noch regelmäßig eine fachspezifische Vertiefung erfolgt.

In der Zahnmedizin ist die Anwendung von EPA in der Ausbildung noch nicht weit verbreitet. Das EPA-Konzept wurde aber sowohl an Zahnkliniken in Nordamerika (University of North Carolina, USA) als auch in Europa (Nijmegen, Niederlande) aufgegriffen. Es wurden Kompetenzkataloge entwickelt, die bis zu 15 für die zahnmedizinische Ausbildung relevante Kompetenzen enthalten. In der Radboud University in Nijmegen wurde für Zahnärzt:innen und Dentalhygieniker:innen eine kompetenzbasierte Unterrichtsform entworfen, in der klinische Tätigkeiten anvertraut werden („entrustment“). Selbstbestimmung und Selbstregulation sollen gefördert und Studierenden die Mitverantwortung für ihren Lernerfolg übertragen werden [5–7].

## Prüfungsformate in der Zahnmedizin

Für viele Lehrveranstaltungen des Studiengangs Zahnmedizin muss eine erfolgreiche Teilnahme nachgewiesen werden. Damit haben diese Lehrveranstaltungen gleichzeitig die Funktion von Hochschulprüfungen. In den betreffenden präklinischen und klinischen Lehrveranstaltungen werden als Formate der praktischen und zum Teil der theoretischen Prüfungen Testate angewendet.

Bei Testaten handelt es sich um eine „Ja/Nein“-Beurteilung einzelner aufeinander aufbauender Arbeitsschritte, die als Kontrollmechanismus gedacht sind, um die selbstständige Durchführung der geleisteten Teilschritte zu dokumentieren, z. B. bei der Anfertigung einer Prothese im praktischen präklinischen Kurs. In den Kursen werden bei der Testatvergabe zudem mündliche Rückmeldungen zur Güte und zu Verbesserungsmöglichkeiten des durchgeführten Arbeitsschrittes erteilt. Diese sind also Teil des Lehrkonzeptes und sollen sicherstellen, dass die durchgeführten Tätigkeiten am Phantommodell und bei der Patientenbehandlung dem erforderlichen Qualitätsstandard entsprechen.

In den praktischen klinischen Kursen wurde in der Vergangenheit für die Attestierung der erfolgreichen Teilnahme ein definierter Katalog an Mindestanforderungen, inklusive aller hierfür erforderlichen Teilschritte, absolviert. Durch die Vergabe der Testate war sichergestellt, dass nur Studierende, die die quantitativen Vorgaben bei der Patientenbehandlung erfüllen, den praktischen klinischen Kurs erfolgreich abschließen können.

Bei der Vergabe von Testaten findet eine Rückmeldung zur Güte des Arbeitsschritts meist in mündlicher Form statt. Die Art und Weise der Rückmeldung ist direkt, unstrukturiert und im Einzelfall wenig ausgewogen. Eine Rückmeldung an Studierende kann somit missverständlich ausfallen, als Kritik aufgefasst und dadurch abgelehnt oder als demotivierend empfunden werden. Die Wirkung der inhaltlichen Rückmeldung des Lehrenden kann in der Folge verloren gehen.

Für die Beurteilung klinischer Tätigkeiten wurde ein EPA-Modell in Anlehnung an die im klinischen Bereich des Regelstudiengangs bestehenden Mindestanforderungen entwickelt. Die EPA finden auch während des „frühen Patientenkontaktes“, also in den Modulen mit ersten klinischen Bezügen, Anwendung; hier werden die Studierenden an die Entwicklung der EPA und deren Bewertung mittels des ABCD-Schemas herangeführt.

Alle Testate wurden im Curriculum iMED DENT im präklinischen und klinischen Bereich durch die Bewertung mittels eines ABCD-Schemas ersetzt, das anstelle einer reinen „Ja/Nein“-Bewertung und mündlich erteilten Rückmeldung die Bewertung verfeinert und verschriftlicht (siehe Abschnitt zum Bewertungsschema unten).

## Aufbau von EPA im Modellstudiengang iMED DENT

### Kompetenzmodell für iMED DENT

Der Begriff „professionelle Kompetenz“ umfasst nach Epstein und Hundert im klinischen Versorgungskontext „den gewohnheitsmäßigen und umsichtigen Einsatz von Kommunikation, Wissen, technischen Fähigkeiten, klinischem Denken, Gefühlen, Werten und Überlegungen in der täglichen Praxis zum Wohle des Einzelnen und der Gemeinschaft, der man dient“ [8]. Ziel des integrierten Modellstudiengangs iMED DENT ist daher, aus den für den Zahnarztberuf relevanten klinischen Tätigkeiten eine repräsentative Auswahl zu treffen, die einen fundierten Start in das Berufsleben sicherstellt. Eine Basis für die Auswahl der zu lehrenden zahnärztlichen Tätigkeiten stellt unter anderem der Nationale Kompetenzbasierte Lernzielkatalog Zahnmedizin (NKLZ) dar [9].

Diese zahnärztlichen Tätigkeiten beinhalten die durchzuführenden Interventionen und als deren Voraussetzung sind theoretische Kenntnisse aus dem zahnmedizinischen Bereich, Grundlagen aus der Medizin mit Bezug zum Patientenkontext und darüber hinaus kommunikative und psychologische Fertigkeiten sowie Grundkenntnisse des wissenschaftlichen Arbeitens erforderlich [10].

Die Vermittlung und Bewertung klinischer Tätigkeiten im Kontext der Ausbildung stellt im Zahnmedizinstudium eine exklusive Situation dar, da die Studierenden direkt an Patient:innen arbeiten. Im zahnmedizinischen Staatsexamen sind die im klinischen Studienabschnitt erworbenen Fähigkeiten bereits Bestandteil der praktischen Prüfungen, die ebenfalls an Patient:innen durchgeführt werden. Für das Curriculum im Modellstudiengang iMED DENT wurde ein Konzept entwickelt, das für die klinische Ausbildung wesentliche Bausteine einer Behandlungssituation als anvertraubare klinische Tätigkeiten (EPA) definiert. Die im integrierten klinischen Unterricht gelehrten Tätigkeiten sind in Tab. 1 zusammengefasst. Diese Tätigkeiten kommen in realen Behandlungssituationen zum Teil verbunden und auch mehrfach vor – beispielsweise sind die Befundaufnahme und Planung Bestandteile jeder Behandlung. Die Auftrennung der einzelnen Tätigkeiten einer klinischen Behandlung in einzelne EPA (beispielsweise Befundaufnahme/Planung/Füllung/festsitzender Zahnersatz) war aufgrund der Operationalisierbarkeit notwendig.

**Table 1:** 

EPA	Zu erbringende Anzahl	Kompetenzstufe
Anamnese, extraoraler und intraoraler Befund, synoptische Befundaufnahme	4	Proficient
Behandlungsplanung	4	Proficient
Direkte mehrflächige Restauration (davon mindestens 2 approximale Seitenzahnfüllungen)	6	Proficient
Endodontische Diagnostik und Therapie	2	Proficient
Prävention, Diagnostik und Therapie	2	Proficient
Parodontale Diagnostik und Therapie	2	Proficient
Festsitzender Zahnersatz (davon optional eine Teilkrone)	4	Proficient
Abnehmbarer Zahnersatz im Lückengebiss (ein Kiefer) Oder Abnehmbarer Zahnersatz bei Zahnlosigkeit (ein Kiefer) Oder Kombiniert festsitzend-abnehmbarer Zahnersatz (ein Kiefer) Oder Provisorischer abnehmbarer Zahnersatz (2 Kiefer)	1	Proficient
Prävention und Erhaltungstherapie bei Senioren	1	Advanced Beginner

*Quelle*: eigene Tabelle

Ein Beispiel für eine zu erlernende klinische Tätigkeit ist die Herstellung einer Krone für einen Zahn. Innerhalb der EPA „Herstellung einer Einzelkrone“ werden verschiedene Kompetenzen erforderlich sein (Tab. 2). Eine Behandlungsstrecke erfordert von der Anamnese und Befundaufnahme (separate EPA) über die Diagnose (tiefe Zerstörung durch Karies) und Prognosestellung bis hin zur Behandlungsplanung jeweils *medizinische, zahnmedizinisch-theoretische und praktische Kenntnisse*. Diese münden im Falle einer Indikation für eine Krone in eine invasive Behandlung, in der eine *zahnmedizinisch-manuelle Fertigkeit* vorliegen muss. Diese stellt innerhalb der EPA „Herstellung einer Einzelkrone“ einen zentralen Anteil dar, der in den früheren Ausbildungsansätzen als alleinige Fertigkeit stellvertretend für die gesamte Einheit „Krone“ bewertet wurde. Im Falle der Herstellung von festsitzendem Zahnersatz (hier Einzelkrone) zählen zur zahnmedizinisch-manuellen Fertigkeit alle Schritte von der Lokalanästhesie über die Kariesentfernung, die Aufbaufüllung, die Zahnpräparation bis zur Eingliederung. Eine *adäquate Kommunikation* mit den behandelten Patient:innen ist in der gesamten Kompetenzkette zwingend nötig. Diese beinhaltet neben der erforderlichen sachlichen Aufklärung auch Elemente, die als „ärztliches Verhalten“ und „Haltung“ eingeordnet werden können. Eine professionelle Form des Umgangs, ein adäquates Angebot zur Patientenpartizipation genauso wie ein empathisches Vorgehen sind erforderlich. Gefühle, Ängste, Schmerzen und atmosphärische Aspekte spielen in einem zahnärztlichen Setting in der Regel eine besondere Rolle.

**Table 2:** 

Kompetenz nach [11]	Medizinisches Expertenwissen	Patientenversorgung	Verhalten und Kommunikation	Professionalität	Praktisches Lernen und kontinuierliche Weiterbildung	Systembezug
*Kompetenzen iMED DENT*	*Theoretische Vorbereitung*	*Praktische Ausführung*	*Ärztliches Verhalten*		Nicht berücksichtigt	Nicht berücksichtigt
*Beispiel: EPA „Herstellung einer Einzelkrone“*	x	x	x		–	–

*Quelle*: eigene Tabelle

*EPA* Entrustable Professional Activities

Für jede EPA der zahnärztlich-klinischen Tätigkeiten haben wir aus dem Kompetenzmodell von ten Cate [11, 12] entsprechend die beschriebenen 3 essenziellen Komponenten verwendet: medizinisches Expertenwissen (theoretisches Wissen), die zahnmedizinisch-manuelle Fertigkeit (praktische Durchführung) sowie eine adäquate Kommunikation (ärztliches Verhalten). Die weiteren Skills aus dem vollständigen Kompetenzmodell von ten Cate wurden als weniger relevant eingestuft und auch im Sinne der Vereinfachung nicht berücksichtigt (Tab. 2).

### Erwerb von Kompetenzstufen

In Abhängigkeit vom Studienfortschritt, also dem jeweiligen Fachsemester, ist ein unterschiedlicher Grad an Supervision für die Durchführung klinischer Tätigkeiten vorgesehen. Um den Grad der Supervision zu bemessen, wurden im iMED DENT 3 *Kompetenzstufen* definiert:

- Advanced Beginner (EPA-Stufe 1): kontinuierliche Supervision,
- Competent (EPA-Stufe 2): engmaschige Supervision,
- Proficient (EPA-Stufe 3): punktuelle Supervision.

Ein Beispiel für den schrittweisen Aufbau der *zahnmedizinisch-manuellen Fertigkeit* ist in Tab. 3 dargestellt. Die angegebenen Zeitpunkte können individuell etwas abweichen. Der Gegenstandskatalog der jeweiligen Fachsemester wird entsprechend dem Kompetenzniveau gestaltet. Hierbei zeigen sich Abweichungen vom Kompetenzaufbau, wie er in der Originalliteratur dargestellt ist [11]. Vor dem Erreichen der Kompetenzstufe EPA 1 werden im integrierten Modellstudiengang iMED DENT bereits intensive Vorbereitungen für den manuellen klinischen Skill getroffen (EPA-Stufe „0“). Parallel zum Aufbau des theoretischen Wissensgerüstes findet ein in der Intensität und Komplexität ansteigendes manuelles Training statt.

**Table 3:** 

Ausbildungsinhalt	Kompetenz	EPA-Stufe	Zeitpunkt
Aufwachsen einer Zahnform	Grundkenntnisse Morphologie und Material	0 (Vorbereitung)	1. Fachsemester, Modul A
Präparation einer Form	Feinmotorisches manuelles Training	0 (Vorbereitung)	1. Fachsemester, Modul B1
Assistenz in der klinischen Behandlung (IK) bei Durchführung der Behandlung durch Studierende höherer Semester	Beobachtung (Supervision nicht vorgesehen)	1 (Advanced Beginner)	5. Fachsemester, Modul F1
Zahnpräparation zur Aufnahme einer Krone am Phantom	Feinmotorisches manuelles Training (intensive, kontinuierliche Supervision)	1 (Advanced Beginner) 2* (Simulation)	3. Fachsemester, Modul D1
Herstellung einer Einzelkrone im klinischen IK „Synoptische Behandlung I“	Durchführung unter Supervision (engmaschige Supervision)	2 (Competent)	7.–10. Fachsemester, Module F2P und G2P
Herstellung einer Einzelkrone im klinischen IK „Synoptische Behandlung II“	Durchführung unter punktueller Supervision	3 (Proficient)	7.–10. Fachsemester, Module F2P und G2P
Einzelkrone in der zahnärztlichen Staatsprüfung Teil 3	Selbstständige Durchführung in Prüfungssituation (Eingreifen nur im Bedarfsfall)	Nicht definiert, da nicht Teil der eigentlichen Ausbildung	Nach Abschluss 10. Fachsemester

*Quelle*: eigene Tabelle

*EPA* Entrustable Professional Activities, *IK* integrierter Behandlungskurs

Im 3. Studienjahr assistieren die Studierenden bei zahnärztlichen Interventionen von Studierenden des 4. oder 5. Studienjahrs. Hier wird erstmals die EPA-Stufe 1 erreicht. Die Phase der Observation wird ergänzt durch die gleichzeitige Simulation am Phantom. In dieser Evolutionsstufe erfolgt die Simulation des Beschleifens von Zähnen im sogenannten Phantomkopf. Dies ist die technisch nächste Annäherung an die Situation im Patientenmund. Dies entspricht aufgrund der weiterhin proaktiven Supervision ebenso dem EPA-Kompetenzlevel 1 – auch weil die reale klinische Situation noch nicht gegeben ist. Parallel werden die begleitenden Kompetenzen wie Befundaufnahme bereits an Patient:innen durchgeführt und gelehrt.

Die erste Kompetenzstufe „Advanced Beginner“ (EPA 1) wird im Rahmen anderer Kompetenzen beispielsweise bei Teilschritten von Behandlungen angewandt. Als Beispiel sei hier die Durchführung der Lokalanästhesie erwähnt. Hierbei findet eine Eins-zu-eins-Betreuung bei der erstmaligen Durchführung einer Lokalanästhesie im 6. Fachsemester (Modul G1), die Studierende gegenseitig durchführen, statt. Auch in den nachfolgenden Fachsemestern 7 und 8 findet weiter eine kontinuierliche Supervision bei der Lokalanästhesie an Patient:innen statt. Erst in den Fachsemestern 9 und 10 wird im Einzelfall je nach Einschätzung durch die Lehrenden entschieden, wie weit der Grad der Supervision hierbei geht. Entsprechend ist die Entwicklung der Kompetenzstufen ein kontinuierlicher Prozess, der nicht in einer harten Stufung abgebildet werden kann.

Die Vorbereitung auf zahnärztlich-invasive Tätigkeiten werden sukzessive weitergeführt, bis im 4. Studienjahr der reale Patientenkontakt um Behandlungen erweitert wird. Eine beispielhafte Darstellung der zu erwerbenden Kompetenzen in der integrierten klinischen Ausbildung ist in Tab. 1 dargestellt. Die Kompetenzen im 4. und 5. Studienjahr unterscheiden sich im Wesentlichen in der zu erbringenden Anzahl. Die engmaschige Supervision beinhaltet die Instruktion auf den verschiedenen relevanten Ebenen, insbesondere der medizinischen Intervention und des ärztlichen Verhaltens. Die lehrenden Zahnärzt:innen unterstützen bei der Durchführung der Zahnpräparation und Abformung – verbal instruktiv, oft auch durch eigene Demonstration des jeweiligen operativen Vorgehens. Gleichzeitig wird das ärztliche Verhalten der Studierenden beobachtet und coachend unterstützt. Jeder Behandlungssitzung geht eine Absprache in Bezug auf die theoretische Vorbereitung voraus – gleichermaßen folgt eine Nachbesprechung, währenddessen Aspekte der Leistungsbewertung diskutiert werden. Entsprechend wird mit dem erfolgreichen Abschluss des 4. Studienjahrs die EPA-Stufe 2 (Competent) erreicht.

Die individuelle Komplexität der Behandlungen stellt einen kaum standardisierbaren Nebeneinfluss auf den angestrebten Kompetenzaufbau dar. Eine Steuerung findet hier in geringem Grad über die Auswahl der Patient:innen statt, wobei in den Fachsemestern 7–8 (4. Studienjahr, integrierter Kurs „Synoptische Behandlung I“) möglichst Patient:innen mit insgesamt geringerem Behandlungsbedarf und in den Fachsemestern 9–10 (integrierter Kurs „Synoptische Behandlung II“) solche mit aufwendigerem Behandlungsbedarf, also z. B. mehr zu behandelnden Zähnen oder einem fortgeschrittenen Schweregrad der Befunde, betreut werden. Unter kontinuierlich reduzierter Supervision wird der klinische Studienabschnitt im 5. Studienjahr fortgeführt. Die Anzahl der Nachweise der jeweiligen EPA wird dabei erhöht, indem die Studierenden Behandlungen häufiger durchführen. Damit gekoppelt soll die Eigenständigkeit bei der Leistungserbringung steigen. Entsprechend wird mit dem Abschluss des 5. Studienjahres das Kompetenzlevel „Proficient“ (EPA-Stufe 3) angestrebt. Bis zum Erreichen der Abschlussprüfung werden die EPA mehrfach wiederholt.

Die zahnärztliche Prüfung Teil 3 (Z3) nach der gültigen ZApprO (ausgefertigt 2019; [2]) stellt in Deutschland die Abschlussprüfung dar. Im Rahmen dieser Abschlussprüfung müssen die Kandidat:innen eine Auswahl der EPA selbstständig umsetzen. Eine unterstützende Intervention durch Lehrpersonal ist nicht mehr vorgesehen. Die Supervisionsfunktion ist daher weitgehend distanziert und auf Interventionen im Fehlerfall reduziert. Diese unabhängige Prüfung betrachten wir daher nicht aus der Sicht des EPA-Kompetenzmodells, da die Staatsprüfungen kein Teil der Ausbildung sind.

### Bewertungsschema für die Kompetenzen innerhalb der EPA zu den unterschiedlichen Zeitpunkten

Die Bereiche *Praktische Durchführung, Theoretisches Wissen* und *Ärztliches Verhalten* der Tätigkeiten/Skills werden separat mittels eines ABCD-Schemas bewertet. Beim ABCD-Schema beschreiben die Buchstaben die Abstufung in der Qualität der erbrachten Tätigkeiten:

- A = einwandfrei (3 Punkte),
- B = befriedigend, mit korrigierbaren Mängeln (2 Punkte),
- C = ungenügend, mit nicht korrigierbaren Mängeln (1 Punkt),
- D = schlecht, Leistung nicht bzw. nicht vollständig erbracht (0 Punkte).

Dieses Schema, das digital, schriftlich dokumentiert wird, ermöglicht eine genauere Bewertung im Gegensatz zur üblicherweise verwendeten Ja/Nein-Testierung.

Die ABCD-Bewertung hat innerhalb der Kompetenzentwicklung 2 Funktionen: (1) das Feedback für die Studierenden, inwieweit sie innerhalb der bewerteten Kompetenz das jeweilige Niveau erreicht haben. Entsprechend sollte mit einer besseren Bewertung (A) ein höheres Maß an Sicherheit und Selbstständigkeit in der Umsetzung einhergehen, gekoppelt an eine reduzierte Intensität der Supervision. (2) Die Bewertung mit ABCD wird in einem Punktesystem verwendet, um das Bestehen des Kurszieles zu ermitteln.

Ein Werkzeug zur Förderung der Selbstreflexion ist die Selbstbewertung der Studierenden unabhängig und zusätzlich zum Lehrenden. Die eigene Leistung wird ebenfalls mittels des ABCD-Schemas bewertet. Anschließend finden ein Abgleich und eine Diskussion der beiden Bewertungen und die finale Dokumentation der abgestimmten Bewertung statt. Hierbei entsteht im Gespräch die Möglichkeit, die Bewertungen zu begründen und Verbesserungspotenziale zu beschreiben, was letzten Endes bei den Studierenden zu einer verbesserten Akzeptanz der Rückmeldung und Anerkennung des eigenen Lernfortschritts führt. Lehrende sind aber frei, ein Ergebnis auch selbstständig festzulegen.

Die ABCD-Bewertungen der Kategorien „Theoretisches Wissen“ und „Klinische Durchführung“ gehen jeweils doppelt gewichtet und „Ärztliches Verhalten“ einfach gewichtet in die Bewertung ein (40 %/40 %/20 %). Die Begründung liegt in der Tatsache, dass Theorie und Durchführung einer klinischen Tätigkeit zwingend vorliegen müssen, um Patient:innen bei der Behandlung keinen körperlichen Schaden zuzufügen, was durch eine exzellente Patientenführung allein nicht gegeben ist. Aus den einzelnen ABCD-Bewertungen der 3 Kategorien wird die Gesamtbewertung der EPA als arithmetisches Mittel gebildet.

## Anwendung von EPA in der klinischen Ausbildung im Modellstudiengang iMED DENT

Die aktuelle ZApprO [2] sieht für die klinische Ausbildung integrierte Behandlungskurse vor. Die Betreuung der Studierenden und Patient:innen findet in den integrierten Behandlungskursen gemeinsam durch die beiden Kernfachdisziplinen statt und schafft dadurch eine praxisnahe Behandlungssituation, in der alle Befunde aus der Therapieplanung berücksichtigt werden. Ziel ist das Erlernen eines synoptischen Behandlungskonzeptes. Bei den Patient:innen findet eine ausführliche allgemeine und spezielle Anamneseerhebung statt. Falls erforderlich, wird zuerst eine Schmerzbehandlung und nachfolgend ausführliche zahnärztliche Befundaufnahme durchgeführt. Die Diagnosestellung, die zahnbezogene Prognose und die Therapieplanung schließen sich an. Die Patient:innen durchlaufen jeweils eine systematische Behandlungsphase auf Grundlage evidenzbasierter Zahnmedizin mit dem Ziel der Rehabilitation des gesamten Kausystems. Jede/r Patient:in erhält nach Abschluss der Therapie ein auf das Risikoprofil zugeschnittenes Nachsorgekonzept.

In der klinischen zahnmedizinischen Ausbildung der Studierenden der 7.–10. Fachsemester sind alle allgemeinzahnärztlichen professionellen klinischen Tätigkeiten als EPA, von der Befunderhebung bis zur Therapie, Teil des Unterrichts. Die EPA leiten sich wie einleitend erklärt von den Tätigkeiten ab, die für den allgemeinzahnärztlichen Beruf erforderlich sind (Tab. 1). Diese beinhalten, vereinfacht zusammengefasst, Zahnhartsubstanzverluste, die mit Maßnahmen zur Vitalerhaltung der Pulpa, Wurzelkanalbehandlungen, direkten und indirekten Restaurationen versorgt werden, sowie die Therapie von Lückengebissen mit festsitzendem oder herausnehmbarem Zahnersatz. Hiervon ausgehend wurde der Ausbildungskatalog für die 4 klinischen Fachsemester festgelegt (Tab. 3). Hier wurde nicht nur die Art der durchzuführenden klinischen Tätigkeiten, sondern auch die zu erbringende Anzahl pro Zeiteinheit eines Jahres vorgegeben. Dieses Vorgehen stellt sicher, dass sich durch die fortschreitende Lernspirale eine Routine und der notwendige Qualitätsstandard der zahnärztlichen Tätigkeiten einstellt. Sie führt dazu, dass nach Abschluss des 10. Fachsemesters das Kompetenzlevel für eine selbstständige Durchführung dieser Tätigkeiten im nachfolgenden beruflichen Einstieg vorliegt.

## Gestaltung des digitalen Testatheftes zur Erfassung der Bewertungen im EPA-Verfahren

**Figure 1:**
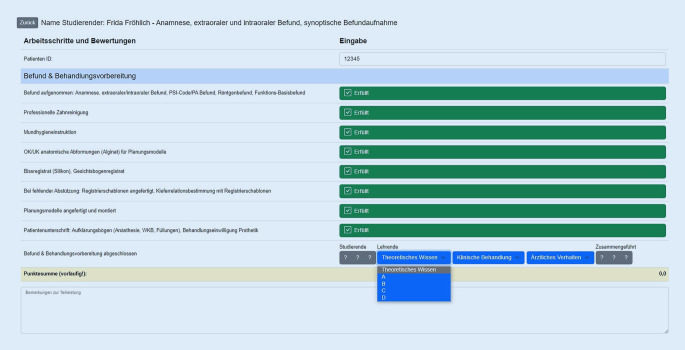

Die Dokumentation von Kursleistungen hat durch die Verwendung von EPA und ABCD-Schema sowie durch die Differenzierung der Bewertung nach den Bereichen „Praktische Durchführung“, „Theoretisches Wissen“ und „Ärztliches Verhalten“ an Komplexität gewonnen. Daher wurde für iMED DENT eine Datenbank zur digitalen Dokumentation entwickelt, die den Prozess der Bewertung und die diesbezügliche Kommunikation zwischen Lehrenden und Studierenden unterstützt. Das digitale Verfahren (Abb. 1) ersetzt die bisherige papierbasierte Dokumentation in Testatheften.

Alle Bewertungen, also auch die Selbstbewertung der Studierenden, fließen im digitalen Testatheft zusammen, der Lernfortschritt ist jederzeit einsehbar und somit transparent. Der Prozess der Aufsummierung aller Kursleistungen findet automatisiert und laufend statt, im Gegensatz zu zeitaufwendigem händischen Auszählen von Papierdokumenten. Eine Beeinflussung durch verschiedene Lehrende kann nicht stattfinden, da der Vorgang standardisiert und teilweise verblindet durchgeführt wird; weiterhin sind Fehlerquellen bei der Auszählung eliminiert. Ebenso kann die Dokumentation nicht verloren gehen und die Archivierung der klassischen Testathefte entfällt.

## Fazit

EPA beschreiben anvertraubare professionelle klinische Tätigkeiten, die zu einem gegebenen Zeitpunkt mit einem gegebenen Kompetenzniveau ausgeübt werden sollen. Da im Zahnmedizinstudium klinische Behandlungen ähnlich dem Niveau einer (postgradualen) Fachweiterbildung ausgebildet werden, lässt sich das Modell der „kompetenzbasierten Ausbildung“ sehr gut auf die zahnärztliche Ausbildung übertragen. Mithilfe einer strukturierten und differenzierten Bewertung (ABCD) der definierten anvertraubaren professionellen Tätigkeiten (EPA) wird die Förderung einer realistischen Selbsteinschätzung der erworbenen Fähigkeiten unterstützt. Ein besonderes Augenmerk erfährt dabei die Selbstbewertung der Studierenden und die Diskussion der Ergebnisse im Kontrast zur Einschätzung der Lehrenden. Gleichzeitig wird mit der Ergänzung des EPA-Modells um ein Bewertungssystem der notwendigen Leistungserfassung innerhalb der Hochschulprüfungen Rechnung getragen.
